# Navigating exercise attitudes and emotional positivity in adulthood during the COVID-19 pandemic: evidence from Shandong Province

**DOI:** 10.3389/fpsyg.2025.1472541

**Published:** 2025-02-14

**Authors:** Ran Meng, Chen Dong

**Affiliations:** ^1^School of Sports Leisure, Shandong Sport University, Jinan, China; ^2^School of Sports Management, Shandong Sport University, Jinan, China

**Keywords:** physical exercise attitudes, emotional positivity, COVID-19 pandemic, demographic influences, health behaviors

## Abstract

**Introduction:**

During the onset of the COVID-19 pandemic, understanding public attitudes and emotional responses towards physical exercise became crucial. This study aimed to explore the relationship between positive emotions and attitudes towards physical exercise among adults in Shandong Province, China, and to identify the influence of demographic factors such as gender, age, and education levels on these attitudes and emotions.

**Methods:**

A cross-sectional study was conducted among 404 adults in Shandong Province, China. Data were collected through surveys that assessed participants’ attitudes towards physical exercise and their emotional positivity. Quantitative analysis was performed to examine the correlations and differences based on demographic variables, including gender, age, and education levels.

**Results:**

A robust positive correlation was found between positive emotions and attitudes towards physical exercise, with an emotional positivity ratio of 2.01±1.38% among respondents. Significant differences in attitudes and emotional positivity were observed based on demographic factors. Specifically, male participants had a higher mean score in behavioral intention and control compared to females. Older adults (>41 years) demonstrated more positive exercise attitudes, with a higher total score of 4.05 ± 0.54 compared to younger age groups. Additionally, respondents with a graduate degree and above reported the highest emotional positivity rate, outpacing those with lower educational levels.

**Discussion:**

The findings indicate that demographic factors, including gender, age, and education levels, play a crucial role in shaping attitudes and emotional responses towards exercise, especially during a global health crisis. These insights highlight the importance of considering demographic differences when promoting physical exercise and designing public health interventions. Future research should further explore the underlying mechanisms and potential interventions to enhance positive attitudes and emotional responses towards physical exercise across diverse populations.

## Introduction

Physical exercise has benefits in many aspects, including physical function, muscle strength, muscle power (Sáez de Asteasu et al., 2019), neuroprotection and cognitive performance (Mollinedo Cardalda et al., 2019), and sleep quality (Hedlund et al., 2019). Physical exercise also has preventive or therapeutic benefits in most chronic diseases and microbial infections (Pedersen and Saltin, 2015). Unfortunately, the COVID-19 pandemic has a tremendous impact on individuals worldwide (Abreu et al., 2024; Marler et al., 2024). In China, it brought challenges to the implementation of the “Healthy China 2030 Action Plan” (Council). The COVID-19 pandemic has caused great inconvenience to daily life, including exercise. Sedentarism has been considered one of the leading causes of disease burden (Lim et al., 2012). The sedentary time has increased during the COVID-19 pandemic period compared with pre-COVID-19 (Jiao et al., 2020). A review suggested that physical exercise is an auxiliary tool for strengthening and preparing the immune system in COVID-19 (da Silveira et al., 2021). All these studies suggest that more attention should be paid to the public’s physical exercise and related factors (Pedrosa et al., 2020).

It is known that emotions and attitudes toward physical exercise may influence the exercise behavior (Padial-Ruz et al., 2020; Padin et al., 2017). The regulation of emotions involves several socio-cognitive factors, the most important of which is attitude (Harmon-Jones et al., 2011; Mauss et al., 2006). Individuals with positive attitudes are inclined to adopt regulation strategies consistent with those attitudes, and are more likely to employ specific regulation strategies (Mauss et al., 2007). Seligman et al. (2004) pointed out that strength and virtue are associated with positivity. The elements of positive mentality proposed by Qian and S (2006) include positive emotions and experience, individual traits, and the social environment. Psychology aims to help people experiencing “adverse conditions” (i.e., anxiety, depression, and interpersonal tension) survive and develop. It also seeks to help people build high-quality social and personal lives to achieve improved happiness, health, and positivity. Fredrickson and Losada (2005) identified the important concepts of positive emotions as joy, gratitude, peace, interest, hope, pride, amusement, encouragement, awe, and love. She noted that positive emotions enhance cognition, attentiveness, creativity, tenacity, subjective well-being, and health.

Many studies have highlighted the importance of physical exercise in health. To prevent a deterioration in their functional capacity, engaging in physical activities can significantly enhance the quality of life and maintain the functional capabilities of seniors. This engagement also supports their ability to independently manage daily living tasks. Conversely, living alone can impact an individual’s basic activities and functional abilities, as the absence of consistent companionship may reduce their incentive to lead an active and healthy lifestyle (Parra-Rizo et al., 2022). When considering the well-being of seniors, it is crucial to identify the factors that contribute to their high satisfaction with their health. The primary determinants of this satisfaction are sustaining optimal functional abilities, being free from physical ailments and mental health issues, and ensuring regular engagement in physical exercise (Agustí et al., 2023).

The “Healthy China 2030 Action Plan” has identified physical exercise as fundamental. However, little is known about the current emotions and attitudes of the public toward physical exercise. Therefore, we performed this study to determine emotions and attitudes toward physical exercise in adults in Shandong province.

## Subjects and methods

### Study design and samples

A cross-sectional study was designed between January 2020 and February 2020. We randomly selected adults who were at least 18 years old in Shandong Province as respondents.

### Questionnaire design

The respondents were surveyed using an exercise attitude questionnaire (Qiwei, 1996; Rongjian, 2003), and a positive emotion self-test (Fredriksen and Wang Mao, 2009) to evaluate the exercise status and emotions. Questionnaires were generated through the Wen juan network^1^ and were sent to the target population through WeChat software.

### Positive emotion self-test

The “Positive Emotion Self-Test” scale from *The Power of Positive Emotions* by Barbara Fredrickson was used to measure positive emotions (Fredriksen and Wang Mao, 2009). This test has 20 questions, covering 10 positive and 10 negative emotions. For positive emotion evaluation, the number of items with a score of 2 or above was recorded for each item as positive emotion items. For negative emotion evaluation, the number of items with a score of 1 or above was recorded for each item as negative emotion items. The positive emotion rate = number of positive emotion items (score ≥ 2)/number of negative emotion items (score ≥ 1). The positive emotion rate ranges from 1 to 3. Higher values indicate greater degrees of positive attitudes. Positivity emotion ratios≥3 suggest the subject may be living well. For other suffering individuals the positivity rate is generally <1.

### Exercise attitude scale

The “Exercise Attitude Scale” compiled by Rongjian (2003) was used to measure attitudes toward physical exercise. There are 70 questions in this scale. It includes eight subscales that measure behavior attitude, goal attitude, behavior cognition, behavior habits, behavior intention, emotional experience, behavior control, and subjective standards (defined below). This scale uses the5-point Likert scoring method, as follows: totally non-conforming (A), non-conforming (B), unclear (C), conforming (D), and fully conforming (E). The scale is scored as follows: A = 1, B = 2, C = 3, D = 4, and E = 5.Reverse scoring items are graded as follows: A = 5, B = 4, C = 3, D = 2, and E = 1. The Cronbach coefficients of each subscale were as follows: behavior attitude = 0.83, target attitude = 0.87, behavior cognition = 0.73, behavior habit = 0.89, behavior intention = 0.84, emotional experience = 0.86, behavior control = 0.80, and subjective standard = 0.64. The internal consistency reliability of each subscale was acceptable. The eight subscales, respectively, represent different aspects of the exercise attitude. Each was scored separately. High scores indicate a normal distribution of the exercise attitude in each dimension and a tendency to have positive attitudes toward physical exercise.

## Definitions

The positive emotion ratio is the positive emotion score divided by the negative emotion score. If the negative emotion is 0, 1 is used instead to avoid situations in which the denominator would be 0 (Fredriksen and Wang Mao, 2009).

Physical exercise attitude refers to the exercise attitude or a person’s understanding of physical exercise and the all-around performance of cognitive process evaluation, emotional experience evaluation, and behavioral intention evaluation during participation in physical exercise (Qiwei, 1996).

The eight subscales of the “exercise attitude scale” are defined as follows (Rongjian, 2003): behavioral habits refer to exercise activities becoming a need of an individual, becoming an automated behavior mode. Target attitude refers to the individual’s positive, negative, or neutral evaluation of exercise at various generalization levels. Behavioral cognition refers to the individual’s actual cognition of a particular result caused by participation in exercise and the evaluation of this cognition. Emotional experience refers to the emotional experience that an individual experiences when participating in exercise or the stimulated emotion when thinking of exercise. Subjective criteria refer to the social pressure perceived by the individual to participate in exercise and the degree of support for participation in exercise by people who influence the individual (parents, elders, close friends, classmates, and idols). Behavioral control refers to the individual’s perception of the difficulty in engaging in exercise behavior and whether they have sufficient autonomy to participate in the exercise. Behavior and attitude refer to an individual’s affirmation, denial, or neutral evaluation of their participation in exercise. Exercise intention refers to whether the individual intends to participate in an exercise, to what extent they are willing to participate in the exercise, and how much effort they plan to make for this.

### Statistical analysis

The SPSS v. 19.0 (IBM Corp., Armonk, NY, United States) software was employed for statistical analysis. The basic characteristics of respondents were analyzed by descriptive statistics. Then, the basic characteristics and differences of the positive emotional rate and the dimensions of exercise attitudes were analyzed by the independent sample *t*-test. Finally, the independent sample *t*-test, analysis of variance, and univariate dispersion analysis were used to test differences in the emotional positive rate and exercise attitudes by gender, age, and educational background.

## Results

### Basic characteristics of respondents

We administered a two-part survey to adults. Completed questionnaires were obtained from 420 of 450 people originally contacted, giving a response rate of 93.33%0.404 valid questionnaires were collected. The respondents were primarily male (249/404, 61.6%), with most in the 31–40-year-old group (186/404, 46.0%). Totally 21.5% of respondents had high school degree and below; 55.7% of respondents had a college or undergraduate degree, and 22.8% had a graduate degree and above (Table 1).

**Table 1:** Basic characteristics of respondents.

Variable	Number (*n* = 404)	Percentage (%)
Gender		
Male	249	61.6
Female	155	38.4
Age, years old		
<31	119	29.5
31–40	186	46.0
>40	99	24.5
Educational background		
High school degree and below	87	21.5
College or undergraduate degree	225	55.7
Graduate degree and above	92	22.8

### Basic characteristics of exercise attitudes and positive emotions

The basic characteristics of exercise attitudes were analyzed. As shown in Table 2, the respondents’ mean score of exercise attitudes was 3.85, corresponding to the upper-middle level. Of all dimensions of exercise attitudes, behavioral cognition scored the highest, with a mean value of 4.60, followed by goal attitude (4.46), emotional experience (4.15), behavioral attitude (4.04), behavioral habit (3.78), behavioral intention (3.42) and behavioral control (3.15); subjective criteria gave the lowest score (2.79).Using descriptive statistics, we also found that the mean score for the positive emotional ratio was 2.01 ± 1.38 (Table 2).

**Table 2:** Analysis results of basic characteristics of exercise attitudes and positive emotion.

Questionnaire	Value (mean ± SD)
Exercise attitudes, average score	3.85 ± 0.61
Behavioral attitude	4.04 ± 0.84
Goal attitude	4.46 ± 0.66
Behavioral cognition	4.60 ± 0.60
Behavioral habit	3.78 ± 0.95
Behavioral intention	3.42 ± 9.78
Emotional experience	4.15 ± 0.83
Behavioral control	3.15 ± 1.01
Subjective criteria	2.79 ± 0.86
Positive emotion assessment	
Number of positive emotion items, n, mean ± SD	7.71 ± 1.75
Number of negative emotion items, n, mean ± SD	4.71 ± 1.67
Positive emotional ratio, mean ± SD	2.01 ± 1.38

### Correlations of exercise attitudes with positive emotions

There were significant positive correlations between attitudes toward exercise and positive emotions, and significant inverse correlations between attitudes toward exercise and negative emotions (Table 3).

**Table 3:** Correlations between exercise attitudes with positive and negative emotions.

Indicator	Positive emotion	Negative emotion
Behavioral attitude	0.420^**^	−0.739^**^
Goal attitude	0.148^**^	−0.601^**^
Behavioral cognition	0.893^**^	−0.269^**^
Behavioral habit	0.791^**^	−0.206^**^
Behavioral intention	0.719^**^	−0.160^**^
Emotional experience	0.840^**^	−0.225^**^
Behavioral control	0.295^**^	−0.315^**^
Subjective criteria	0.133^**^	−0.192^**^

^**^When the confidence level (double test) is 0.01, it indicates a significant correlation.

### Differences in exercise attitudes and positive emotions between genders

The independent sample *t*-test was employed to analyze the differences in the positive emotional rate between genders (Table 4). The mean emotional positive rate of male respondents was 1.80, and that of female respondents was 2.36. There was a significant difference between the two groups (*t* = −3.658, *p* < 0.001). The effect sizes of both independent *t*-tests and ANOVAs were measured by statistical magnitude of Cohen’s *d*, and they were all large than 0.8 in combination with larger sample size, indicating the test results were also valid in actual scenarios.

**Table 4:** Differences in exercise attitudes and positive emotions between genders.

Exercise attitudes	Male (*n* = 249)	Female (*n* = 155)	*p*
	Mean ± SD	Mean ± SD	
Behavioral attitude	4.06 ± 0.84	4.00 ± 0.85	0.482
Goal attitude	4.48 ± 0.69	4.43 ± 0.62	0.436
Behavioral cognition	4.59 ± 0.64	4.61 ± 0.53	0.713
Behavioral habit	3.89 ± 0.92	3.62 ± 0.96	0.005^**^
Behavioral intention	3.50 ± 0.78	3.29 ± 0.77	0.008^**^
Emotional experience	4.19 ± 0.83	4.09 ± 0.83	0.234
Behavioral control	3.23 ± 1.03	3.02 ± 0.96	0.035^*^
Subjective criteria	2.79 ± 0.88	2.80 ± 0.82	0.945
Total score	3.89 ± 0.61	3.78 ± 0.60	0.062
Emotional positive ratio	1.80 ± 1.05	2.36 ± 1.73	0.000^***^

^*^ indicates *p* < 0.05, ^**^ indicates *p* < 0.01, and ^***^ indicates *p* < 0.001.

The independent sample *t*-test was employed to analyze the differences in exercise attitudes between genders (Table 4). Male respondents scored a mean value of 3.89 for exercise attitudes. The mean values for behavioral cognition, goal attitude, emotional experience, and behavioral attitude were greater than or equal to that of exercise attitudes, suggesting a positive effect. Conversely, the mean values for behavioral intention, behavioral control, and subjective criteria were lower than that of exercise attitudes, suggesting a negative effect.

Female respondents scored a mean of 3.78 for exercise attitudes. The mean values for behavioral cognition, goal attitude, emotional experience, and behavioral attitude were higher than that of exercise attitudes, suggesting a positive effect. Conversely, the mean values of behavioral habit, behavioral intention, behavioral control, and subjective criteria were lower than that of exercise attitudes, suggesting a negative effect.

### Differences in exercise attitudes and positive emotions among respondents of different ages

The differences in the positive emotional rate among respondents of different ages were tested by univariate dispersion analysis (Table 5). The mean emotional positive rate of respondents aged below 31 was 2.01; those of respondents aged between 31 and 40 and >41 years were 2.09 and 1.86, respectively. The *F*-value was 0.883, indicating no statistical significance. This finding indicated that there was no significant difference among respondents of different ages.

**Table 5:** Differences in exercise attitudes and positive emotions among respondents of different ages.

Exercise attitudes	<31 years old (*n* = 119)	31–40 years old (*n* = 186)	>41 years old (*n* = 99)	*p*
	Mean ± SD	Mean ± SD	Mean ± SD	
Behavioral attitude	3.96 ± 0.89	3.95 ± 0.85	4.30 ± 0.70	0.001^**^
Goal attitude	4.35 ± 0.76	4.44 ± 0.66	4.64 ± 0.50	0.004^**^
Behavioral cognition	4.55 ± 0.66	4.61 ± 0.56	4.65 ± 0.59	0.452
Behavioral habit	3.73 ± 0.97	3.63 ± 0.95	4.14 ± 0.82	0.000^***^
Behavioral intention	3.49 ± 0.79	3.25 ± 0.76	3.63 ± 0.75	0.000^***^
Emotional experience	4.12 ± 0.91	4.10 ± 0.79	4.28 ± 0.79	0.172
Behavioral control	3.15 ± 1.02	2.95 ± 0.95	3.52 ± 1.03	0.000^***^
Subjective criteria	2.82 ± 0.93	2.77 ± 0.78	2.81 ± 0.92	0.882
Total score	3.82 ± 0.66	3.76 ± 0.58	4.05 ± 0.54	0.000^***^
Emotional positive rate	2.03 ± 1.42	1.83 ± 1.07	2.31 ± 1.76	0.000^**^

^**^ indicates *p* < 0.01, and ^***^ indicates *p* < 0.001.

The differences in exercise attitudes among respondents of different ages were tested by analysis of variance (Table 5). Older individuals tended to have better behavioral attitudes, goal attitudes, behavioral habits, behavioral intention, behavioral control, and total score. There were no significant differences among age groups in behavioral recognition, emotional experience, or subjective criteria.

### Differences in exercise attitudes and positive emotions among respondents with different educational backgrounds

The differences in the positive emotional rate among respondents with different educational backgrounds were tested by univariate dispersion analysis (Table 6). Respondents with higher levels of education had higher emotional positive rates based on the *post hoc* test, while there was no significant difference in the positive emotional rate between respondents with a high school degree or below and those with other educational backgrounds.

**Table 6:** Differences in the positive emotional rate of respondents with different educational backgrounds.

Exercise attitudes	High school degree and below (*n* = 87)	College or undergraduate degree (*n* = 225)	Graduate degree and above (*n* = 92)	*p*
	Mean ± SD	Mean ± SD	Mean ± SD	
Behavioral attitude	4.10 ± 0.89	4.03 ± 0.82	3.99 ± 0.84	0.665
Goal attitude	4.47 ± 0.76	4.47 ± 0.61	4.44 ± 0.70	0.924
Behavioral cognition	4.54 ± 0.72	4.61 ± 0.56	4.63 ± 0.56	0.554
Behavioral habit	4.04 ± 0.99	3.70 ± 0.93	3.75 ± 0.90	0.016^*^
Behavioral intention	3.64 ± 0.79	3.33 ± 0.80	3.41 ± 0.70	0.008^**^
Emotional experience	4.27 ± 0.89	4.10 ± 0.83	4.16 ± 0.76	0.248
Behavioral control	3.45 ± 1.07	3.09 ± 1.01	3.02 ± 0.92	0.005^**^
Subjective criteria	3.00 ± 0.95	2.73 ± 0.83	2.75 ± 0.82	0.043^*^
Total score	3.99 ± 0.67	3.81 ± 0.59	3.82 ± 0.57	0.058
Emotional positive rate	2.03 ± 1.42	1.83 ± 1.07	2.31 ± 1.76	0.000^**^

^*^ indicates *p* < 0.05, ^**^ indicates *p* < 0.01.*Post hoc* test for Emotional positive ratio: respondents with a college or undergraduate degree < respondents with a graduate degree and above.

The differences in exercise attitudes among respondents with different educational backgrounds were tested by analysis of variance (Table 6). Respondents with a high school degree and below scored a mean of 3.99 for exercise attitudes. The mean values for behavioral cognition, goal attitude, emotional experience, behavioral attitude, and behavioral habits were higher than that of exercise attitudes, displaying a positive effect. Conversely, the mean values of behavioral intention, behavioral control, and subjective criteria were lower than that of exercise attitudes, suggesting a negative effect.

Respondents with a college or undergraduate degree scored a mean of 3.81 in exercise attitudes. The mean values for behavioral cognition, goal attitude, emotional experience, and behavioral attitude were higher than that of exercise attitudes, suggesting a positive effect. Conversely, the mean values for behavioral habit, behavioral intention, behavioral control, and subjective criteria were lower than that of exercise attitudes, suggesting a negative effect.

Respondents with a graduate degree and above scored a mean of 3.82 in exercise attitudes. The mean values for behavioral cognition, goal attitude, emotional experience, and behavioral attitude were higher than that of exercise attitudes, suggesting a positive effect. Conversely, the mean values of behavioral habit, behavioral intention, behavioral control, and subjective criteria were lower than that of exercise attitudes, suggesting a negative effect.

## Discussion

Physical exercise has many benefits for health. It is known that emotions and attitudes toward physical exercise may influence the exercise behavior. Therefore, we investigated the basic characteristics of exercise attitudes and positive emotions in adults. Our results suggested that most respondents had positive emotions. Positive emotions were also positively correlated with exercise attitudes. Our findings provide a theoretical reference value for physical exercise. The public should be in a positive mood, with an optimistic attitude into physical exercise.

We found that respondents thought highly of the effect of exercise, and the vast majority of them were willing to participate, loved to do physical exercise, and regarded exercise as necessary. This might be associated with the effects of emotional stability, physical health, family harmony, social stability, and national development on exercise attitude. Another possible reason is that the public’s understanding of physical exercise is relatively adequate, realizing the various benefits of exercise. The absence of significant differences in the positive emotional rate across different age groups suggests that emotional positivity is not the sole determinant of exercise behavior. However, the higher total score for exercise attitudes observed in older adults implies a potential behavioral disparity. This group may exhibit greater consistency in exercise participation due to various factors such as health consciousness and life experience, which could reinforce the importance of tailored exercise interventions that cater to the cognitive and emotional nuances of different age demographics. The non-significant emotional rate differences also underscore the need for a holistic approach to understanding exercise behavior, where emotional, cognitive, and behavioral elements are considered collectively.

Respondents had relatively low scores in voluntary participation in exercise, selection of exercise content, implementation of exercise plans, and control of external factors. A small number of respondents had low emotional positivity. Those performances should be noticed. Avolio stated that interpersonal relations and the sense of organizational support have direct impacts on positive psychological and physiological results, thereby stimulating and enhancing overall psychological potential (Avolio, 2005). Another study found that exercise attitudes produce a direct effect on exercise behaviors and act as a mediating variable to produce an indirect effect on exercise behaviors (Berry et al., 2016).

The positive emotional ratios of most respondents were high, while a few had low rates of emotional positivity. Mauss et al. (2007) probed the effects of control-type emotion regulation attitudes and expression-type emotion regulation attitudes on the use of emotion regulation strategies. They found that in addition to affecting the use of emotion regulation strategies, emotion regulation attitudes are also associated with emotional experiences and physiologically active states.

Women reported more positive emotions than men, but less positive attitudes toward exercise. There were no significant differences in behavioral and goal attitudes, behavioral recognition, emotional experience, and subjective criteria. The mean values for behavioral habit, behavioral intention, and behavioral control were higher in men compared with women. Men had more positive exercise attitudes than women. It may be that, due to the traditional gender culture, with “males working outside and females taking care of home” (Wang and Tang, 2020), women tend to do more housework than men and coached children in their studies, so their attitudes toward exercise might be reduced. Because of pressure arising from low income and other issues, especially during the COVID-19 pandemic, men may have reduced their positive emotions. To relieve stress and regulate emotions, men might tend to perform physical exercise in the context of sufficient spare time. The observed gender differences in emotional positivity and attitudes toward exercise may be deeply rooted in cultural and social norms. Traditional gender roles often portray men as more active and physically robust, which could enhance their positive attitudes and emotional positivity toward exercise. Conversely, women may be influenced by societal expectations that indirectly discourage vigorous physical activity, potentially leading to less positive attitudes toward exercise. The higher positive emotional rate among women, despite their less positive attitudes toward exercise, could be attributed to their ability to find satisfaction in other life domains, highlighting the complex interplay between emotions, culture, and exercise behavior. These findings underscore the importance of developing gender-sensitive exercise interventions that consider the influence of cultural and social norms on emotional positivity and attitudes toward physical activity.

In an exercise scenario, the health belief model holds that people often do not participate in healthy physical exercise unless they have certain levels of exercise motivation, health motivation, and health knowledge, or they believe they are vulnerable to health problems. They may believe their environment poses a threat to their health or believe that physical exercise is beneficial to overcoming their problems. Finally, it is not difficult for them to perform physical exercise (Jian et al., 2016). McAuley et al. (1995) found that after a high-intensity exercise task, respondents’ emotions became increasingly positive, and their belief in completing such tasks in the future was also enhanced.

A possible reason for this finding is that changes in positions and roles in families tend to increase with age. Adults require healthy bodies to bear and raise children and care for the elderly. Only by working and living with positive emotions can they accomplish the missions entrusted to them. Another possible reason is their perception of life and health literacy. We found no difference in the positive emotional rate among respondents of different ages; indeed, there was no difference in positive emotions among respondents of different ages. The mean value for exercise attitudes in respondents older than 41 was significantly higher than those of individuals younger than 31 and individuals between 31 and 40. In terms of the activity evaluation of exercise, evaluation of exercise, automation degree of exercise, willingness to exercise, and cognition of exercise self-control ability, respondents older than 41 scored higher than respondents younger than 31 and individuals aged between 31 and 40.

Groups with distinct educational backgrounds may have different understandings of the meaning of physical exercise (Ridder et al., 2018). These observations might be related to differences in the amount of knowledge reserve, the depth of understanding, and outlooks on life and values. It might also be attributable to cognition of the pandemic or their understanding of information and public opinions in exceptional times. We found that respondents with a graduate degree and above had the highest emotional positive rate, followed by individuals with a high school degree and below and those with a college or undergraduate degree. There are multidimensional factors of positive emotions in respondents with a graduate degree and above. In addition to advanced academic knowledge, these individuals scientifically understand physical exercise, effective execution of virus defense, yearning for a happy life, and cherishing life. Under the normal circumstances of pandemic prevention and control, the mean value for exercise attitudes of respondents with a high school degree and below was higher than that of respondents with a college or undergraduate degree and those with a graduate degree and above. Specifically, they were more willing to exercise. They had more autonomy to participate in exercise. They were more easily affected by people with significant influence around them (such as fitness instructors on TV or online), and they felt tremendous social pressure when participating in exercise. Cherubini stated that positive psychology and physical education teaching are consistent with promoting positive development, and applying positive psychology to physical education teaching allows students to experience creative and meaningful experiences (Cherubini, 2009).

There were some limitations to this study. We only focused on differences in gender, age, and educational background concerning emotional positivity rate and exercise attitude. In addition, we did not determine whether gender, age and education level were independent risk factors for emotional positivity and exercise attitude. More data are needed for determining the nature and interpretation of changes in exercise attitudes and emotions. Compared to similar studies, the strengths of this research include its regional focus during a specific time period—the onset of the COVID-19 pandemic—which provides unique data on adult attitudes and emotions. The comprehensive assessment tools and rigorous statistical analysis on a robust sample size also enhance the study’s credibility and applicability of its findings.

## Conclusion

While this study provides valuable insights into the relationship between emotional positivity and attitudes toward exercise during the COVID-19 pandemic among adults in Shandong Province, it is essential to recognize the limitations in over-generalizing these findings to other populations or contexts. The results are representative of the unique demographic and cultural backdrop of the study population and may not be extrapolated to all adults or to different geographical or cultural settings. Future research should aim to replicate these findings in diverse populations to better understand the universality of the observed relationships between demographic factors, emotional positivity, and exercise attitudes.

This research underscores the theoretical significance for the scientific community by demonstrating the impact of demographic variables on exercise-related attitudes and emotional reactions. It underscores the pivotal role that positive emotions play in driving health-related actions and stresses the importance of integrating cognitive and emotional regulation into models of exercise behavior. Furthermore, the study sheds light on the dynamics of exercise attitudes and emotions during a pandemic, offering a foundation for advancing theoretical frameworks in the domains of health psychology and behavioral change.

The findings reveal a symbiotic relationship between positive emotions and exercise attitudes among adults. It is essential for individuals to approach physical activity with a positive and hopeful mindset, which not only fosters a healthy lifestyle but also equips them to confront global health challenges with resilience and mental well-being.

## Data Availability

The original contributions presented in the study are included in the article/supplementary material, further inquiries can be directed to the corresponding author.

## Footnotes

^1^ https://www.wenjuan.com/
